# Passive or active drainage system for chronic subdural haematoma—a single-center retrospective follow-up study

**DOI:** 10.1007/s00701-024-05967-6

**Published:** 2024-02-19

**Authors:** Paulina Majewska, Mattis A. Madsbu, Lisa Millgård Sagberg, Sasha Gulati, Asgeir Store Jakola, Ole Solheim

**Affiliations:** 1 Department of Neurosurgery, St. Olav’s University Hospital, Trondheim, Norway; 2 Department of Neuroscience, Norwegian University of Science and Technology, Trondheim, Norway; 3 Department of Public Health and Nursing, Norwegian University of Science and Technology, Trondheim, Norway; 4 Institute of Neuroscience and Physiology, Section of Clinical Neuroscience, Sahlgrenska Academy, Gothenburg, Sweden

**Keywords:** Chronic subdural haematoma, Recurrence rate, Subdural drain, Subgaleal drain

## Abstract

**Background:**

Postoperative drainage systems have become a standard treatment for chronic subdural hematoma (CSDH). We previously compared treatment results from three Scandinavian centers using three different postoperative drainage systems and concluded that the active subgaleal drainage was associated with lower recurrence and complication rates than the passive subdural drainage. We consequently changed clinical practice from using the passive subdural drainage to the active subgaleal drainage.

**Objective:**

The aim of the present study was to assess a potential change in reoperation rates for CSDH after conversion to the active subgaleal drainage.

**Methods:**

This single-center cohort study compared the reoperation rates for recurrent same-sided CSDH and postoperative complication rates between patients treated during two study periods (passive subdural drainage cohort versus active subgaleal drainage cohort).

**Results:**

In total, 594 patients were included in the study. We found no significant difference in reoperation rates between the passive subdural drain group and the active subgaleal drain group (21.6%, 95% CI 17.5–26.4% vs. 18.0%, 95% CI 13.8–23.2%; *p* = 0.275). There was no statistical difference in the rate of serious complications between the groups. The operating time was significantly shorter for patients operated with the active subgaleal drain than patients with the passive subdural drain (32.8 min, 95% CI 31.2–34.5 min vs. 47.6 min, 95% CI 44.7–50.4 min; *p* < 0.001).

**Conclusions:**

Conversion from the passive subdural to the active subgaleal drainage did not result in a clear reduction of reoperation rates for CSDH in our center.

## Introduction

Although management of chronic subdural haematoma (CSDH) has evolved over the past decades, recurrence rates still remain high [6]. The most common surgical techniques are drainage via a twist-drill craniostomy or a burr hole. Postoperative drainage systems have become a standard treatment after evidence of reduced recurrence rates and mortality in a multicenter trial [14]. However, the type of drainage varies across centers. Although still controversial, a pooled study from two clinical trials suggested that active drainage is associated with higher recurrence rates [5]. In contrast, we previously compared treatment results from three Scandinavian centers using three different postoperative drainage systems (continuous irrigation and drainage, passive subdural drainage, and active subgaleal drainage) and concluded that the active subgaleal drainage was associated with lower recurrence and complication rates [15]. We consequently changed clinical practice from using a passive subdural drainage to an active subgaleal drainage. The aim of the present study was to assess a potential change in reoperation rates for CSDH after conversion to the active subgaleal drainage.

## Methods

### Patient population

All consecutive patients 18 years or older treated with an evacuation of primary CSDH at St. Olav’s University Hospital in Trondheim, Norway, in the time period January 1, 2005, to December 31, 2010 (the passive subdural drain cohort), and in the time period March 1, 2017, to August 31, 2022 (the active subgaleal drain cohort), were identified. The passive subdural drain cohort has previously been identified and investigated as part of our previous study [15]. The time gap between the inclusion periods represents the time that was required to publish the previous study and change the clinical practice from using a passive subdural drainage to an active subgaleal drainage in our institution. Patients were identified through hospital electronic records and operation logs. The neurosurgical department at St. Olav’s University Hospital serves exclusively a defined geographical catchment region with approximately 750,000 inhabitants. Electronic medical records of all seven local hospitals in the region were available for review. Patients were considered eligible for inclusion if they had been operated with a single or double burr hole with placement of a passive subdural or an active subgaleal drainage system with a minimum of 6-month follow-up. We excluded patients who had undergone previous or intercurrent intracranial surgery. Six patients were excluded from the latest inclusion period because they were operated with a subdural drain instead of a subgaleal drain during the second treatment period.

### Treatment regimens

Both cohorts were treated with a single burr hole and irrigation intraoperatively. The passive subdural drain or the active subgaleal drain was placed at the end of surgery and kept overnight, usually for 12–18 h. All patients were typically observed at an intermediate/postoperative ward unit 2–4 h postoperatively, after which they were transferred to a regular ward unit. The passive subdural drains were kept at the hight of the head, and patients were most often placed on bedrest. The patients treated with active subgaleal drains had no restrictions in regard to drain position or mobilization. Apart from changing the type of postoperative drainage technique and, consequently, mobilization restrictions postoperatively, no other changes were introduced between the two inclusion periods.

### Study variables

We defined the index operation as the first surgical procedure on the affected side. Reoperation for a CSDH recurrence was defined as reoperation for recurrent same-sided CSDH in patients treated within 6 months of the index operation. In patients with bilateral haematomas who had been operated on one side only during the initial operation and later receiving bilateral hematoma evacuation, the event was registered as one index operation and one reoperation. We used electronic medical records to retrospectively retrieve patient and treatment characteristics. Radiological imaging and variables were reviewed using the hospital’s imaging and archiving systems.

### Primary and secondary outcomes

The primary outcome was the proportion of patients that had undergone a reoperation for recurrent same-sided CSDH within 6 months from the initial operation. Secondary outcomes were overall survival, perioperative mortality and surgical complications (within 30 days from the initial operation), analyzed according to the Landriel Ibanez classification [12], and operating time. Operating time data was not available for patients that were included before 01.01.2007.

### Ethics and approvals

The study protocol was evaluated by the Regional Committee for Medical and Health Research Ethics in Central Norway (REK). The study was classified as a retrospective quality control study by the committee, and an ethical approval from REK was deemed unnecessary.

### Sample size calculation

The reoperation rate was used as the outcome variable for the sample size calculation. A previous consecutive population-based comparative cohort study showed that reoperation rate was 20.0% and 11.1% for patients treated with the passive subdural drain and the active subgaleal drain, respectively [15]. Based on this data, a sample size of 520 patients (260 patients in each group) was needed to achieve 80% power, with two-sided significance level of *p* = 0.05.

### Statistical analysis

Statistical analyses were performed using IBM SPSS Statistics for Windows, Version 32 27.0.1.0. Normal distribution of continuous variables was assessed with Q-Q plots. Chi-square test was used in analysis of categorical data. Two-sample two-tailed *t*-test was used in analysis of normally distributed continuous data. Mann–Whitney *U* test was used in analysis of ordinal or continuous data with non-normal distribution. A logistic regression model was used to investigate the potential predictor factors for reoperation. Cases with missing data were excluded from the regression analysis. Statistical significance was set to *p* < 0.05.

## Results

Among the 594 patients that were included in the study, 333 patients were operated with a passive subdural drain and 261 patients with an active subgaleal drain. As seen in Table 1, patient groups were comparable with regard to age, sex, preoperative need for social care, preoperative GCS, side of surgery, the preoperative Charlson Comorbidity Index (CCI), and preoperative use of antiplatelet drugs and anticoagulation. There was, however, a significant difference in the preoperative modified ranking scale (mRS) between the groups. More patients in the passive subdural drain cohort had higher mRS than patients in the active subgaleal drain cohort, indicating lower functional levels in the first cohort. In addition, the passive subdural drain cohort had somewhat smaller hematomas (mean 21.0 vs. 24.2 mm in diameter; *p* < 0.001), as well as more patients with a hypodense hematoma on brain CT compared with the active subgaleal drain cohort.

**Table 1:** Patient characteristics

	Passive subdural drain cohort		Active subgaleal drain cohort		*p*-value
*N*	333		261		-
Age (median/range)	78 (26–96)		77 (20–96)		0.985
Sex	Female	83 (25%)	Female	68 (26%)	0.754
	Male	250 (75%)	Male	193 (74%)	
Premorbid functional level	Independent	237 (71%)	Independent	201 (77%)	0.138
	Home with care	62 (19%)	Home with care	33 (13%)	
	Nursing home	33 (10%)	Nursing home	27 (10%)	
	Unknown	1 (0%)	Unknown	0 (0%)	
Charlson comorbidity index (median/range)	4 (0–12)		4 (0–12)		0.206
Preoperative modified ranking scale	0	2 (1%)	0	6 (2%)	< 0.001
	1	33 (10%)	1	23 (9%)	
	2	74 (22%)	2	70 (27%)	
	3	46 (14%)	3	118 (45%)	
	4	138 (41%)	4	26 (10%)	
	5	40 (12%)	5	18 (7%)	
	6	0 (0%)	6	0 (0%)	
Preoperative GCS (median/range)	15 (3–15)		15 (3–15)		0.218
Number of patients that used antiplatelets preoperatively	94 (28%)		46 (18%)		0.282
Number of patients that used anticoagulation preoperatively	67 (20%)		46 (18%)		0.442
Haematoma diameter in mm (mean/SD)	21.0 (5.7) (46 unknown)		24.2 (5.7) (3 unknown)		< 0.001
Midline shift (mean/SD)	7.5 (4.8) (42 unknown)		8.0 (5.0) (3 unknown)		0.196
Haematoma density	Hypodense	94 (28%)	Hypodense	17 (7%)	< 0.001
	Mixed	125 (37%)	Mixed	169 (65%)	
	Isodense	45 (14%)	Isodense	69 (26%)	
	Hyperdense	13 (4%)	Hyperdense	3 (1%)	
	Unknown	56 (17%)	Unknown	3 (1%)	
Side of surgery	Right	116 (35%)	Right	99 (38%)	0.952
	Left	133 (40%)	Left	108 (41%)	
	Bilateral	66 (20%)	Bilateral	53 (20%)	
	Unknown	18 (5%)	Unknown	1 (1%)	

*SD*, standard deviation; *mm*, millimeter; *GCS*, Glasgow Coma Scale

Seventy-two patients (21.6%, 95% CI 17.5–26.4%) were reoperated in the passive subdural drain group, while 47 patients (18.0%, 95% CI 13.8–23.2%) were reoperated in the active subgaleal drain group, *p* = 0.275. As presented in Table 2, almost all patients in both groups were reoperated due to both clinical and radiological deterioration (92% in both cohorts, *p* = 0.960).

**Table 2:** Documented reasons for reoperations

	Subdural drain (*n* = 72)	Subgaleal drain (*n* = 47)
Clinical deterioration	4 (5%)	3 (6%)
Radiological deterioration	2 (3%)	1 (2%)
Both	66 (92%)	43 (92%)

Of all included patients, 476 had a record of operating time (216 patients with the passive subdural drain and 260 patients with the active subgaleal drain). The proportion of patients that underwent bilateral hematoma evacuation was similar in both groups (subdural 15% vs. subgaleal 20%, *p* = 0.149). We found that the operating time was significantly shorter for patients operated with the active subgaleal drain than patients with the passive subdural drain (32.8 min, 95% CI 31.2–34.5 min vs. 47.6 min, 95% CI 44.7–50.4 min; *p* < 0.001).

Table 3 shows postoperative surgical complications, assessed using the Landriel Ibañez classification scale. There was no statistical difference in the number of patients that experienced serious complications after treatment (grade 3 or 4 complications) between the groups (8 patients in the subdural drain cohort vs. 4 patients in the subgaleal drain cohort, *p* = 0.455). Table 4 shows the registered complications for both cohorts.

**Table 3:** Incidence of postoperative complications

Landriel Ibañez grade	Subdural drain (*n* = 333)	Subgaleal drain (*n* = 261)
Grade 1	14 (4%)	22 (8%)
Grade 2	3 (1%)	6 (2%)
Grade 3	4 (1%)	0 (0%)
Grade 4	4 (1%)	4 (2%)

**Table 4:** Postoperative complications

Complication type	Number of patients	
	Subdural drain (*n* = 333)	Subgaleal drain (*n* = 261)
Epilepsy	2	7
Acute subdural haematoma	5	0
Other intracranial haemorrhage	3	1
Cerebral infarct	0	2
Subdural empyema	1	3
Meningitis	1	0
Wound infection	0	2
Transient ischemic attack	1	1
Pneumonia	1	4
Respiratory failure	1	0
Pulmonary embolism	1	1
Cardiac arrythmias	2	1
Urinary tract infection	6	10
Electrolyte impairment	1	0

In a multiple logistic regression model, the type of postoperative drainage system, age, preoperative functional status (mRS), the use of antithrombotic drugs, haematoma diameter, haematoma density, and haematoma laterality were not significant predictor factors for recurrent surgery for CSDH (Table 5). However, men were more likely to undergo a recurrent surgery for CSDH than women (OR 2.029, 95% CI 1.180–3.489; *p* = 0.010).

**Table 5:** Predictor factors for CSDH reoperation

	Univariable analysis		Multivariable analysis (*n* = 370)	
Factor	Odds ratio (95% CI)	*p*-value	Odds ratio (95% CI)	*p*-value
Age at treatment (continuous)	0.999 (0.983–1.015)	0.869	1.006 (0.988–1.026)	0.507
Sex				
- Female - Male	1 (reference) 1.885 (1.120–3.174)	0.017	1 (reference) 2.029 (1.180–3.489)	0.010
Preoperative modified ranking scale (ordinal)	1.046 (0.882–1.240)	0.604	1.041 (0.860–1.260)	0.681
Use of antithrombotic drugs				
- No - Yes	1 (reference) 0.771 (0.513–1.159)	0.211	1 (reference) 0.673 (0.436–1.039)	0.074
Type of drainage				
- Passive subdural - Active subgaleal	1 (reference) 0.796 (0.529–1.199)	0.275	1 (reference) 0.809 (0.516–1.270)	0.357
Haematoma diameter				
- Below 20 mm - 20 mm or above	1 (reference) 0.729 (0.473–1.121)	0.150	1 (reference) 0.693 (0.437–1.099)	0.119
Haematoma laterality				
- Unilateral - Bilateral	1 (reference) 1.442 (0.949–2.192)	0.087	1 (reference) 1.371 (0.890–2.112)	0.152
Haematoma density				
- Hypodense or isodense - Hyperdense or mixed	1 (reference) 1.038 (0.694–1.553)	0.854	1 (reference) 1.160 (0.757–1.778)	0.496

## Discussion

In our previous multicenter regional comparison study comparing three different drainage techniques for CSDH, we found that the center using the active subgaleal drainage had 50% lower recurrence rates than in our center where the passive subdural drainage was used [15]. However, the subsequent conversion from the passive subdural to the active subgaleal drainage did not result in a clear reduction of recurrence rates in our center. This suggests that the difference between the active and passive drainage may be small. In addition, the current study exemplifies that comparisons across hospitals of the endpoints subjective to judgement, such as indication for reoperations, may be biased. In such analyses of clustered data, the “bundles of care” and local practices may be more important than the investigated intervention. As a result, reoperation rate may not be a valid endpoint in non-randomized multicenter CSDH studies or serve as a good marker of a procedure effectiveness.

The previous literature on the effect of the postoperative drainage type on the recurrence rate of CSDH is rather conflicting. The only multicenter randomized controlled trial (RCT) to date showed lower recurrence rate in patients with subperiosteal active drain compared to patients with passive subdural drain (8.3%, 95% CI 4.3–14.7 vs. 12.0%, 95% CI 6.7–19.7) [16]. This non-inferiority trial concluded that subperiosteal drains are not inferior to subdural drains in regard to recurrence rates for CSDH. Similarly, to the RCT, our previous observational study showed lower recurrence rate with the use of active subperiosteal drains compared to subdural drains [15]. On the other hand, others showed no difference or lower recurrence rates with the use of subdural drains [2, 7, 8, 10, 18]. Three recent systematic reviews and meta-analyses showed that the use of subperiosteal drains is associated with a lower recurrence rate than the use of subdural drains (11.9% vs. 12.3%, RR 0.8, 95% CI 0.67–0.97, *p* = 0.02 [9]; OR 0.73, 95% CI 0.58–0.92, *p* = 0.07 [4], and 12% vs. 12.7%, OR 0.72, 95% CI 0.57–0.91, *p* = 0.005 [17]). However, after excluding our previous study [15] that was the single most influential study in all reviews, the pooled analysis showed no significant difference in recurrence rates between the groups (12.8 vs. 10.5%, RR 0.95, 95% CI 0.74–1.23, *p* = 0.71 [9] and OR 0.94, 95% CI 0.70–1.28, *p* = 0.71 [4]) in two reviews [4, 9]. The same analysis was not performed in the third review, but the results would have most likely been the same [17].

Complication rate associated with using different postoperative drainage types is another important aspect that should be considered. Our current study showed no significant difference in complication rates following surgery with the active subgaleal drainage compared to the passive subdural drainage. These results are in line with our previous study [15] and other similar studies [8, 18]. Nevertheless, there are other studies that showed lower complication rates associated with the active subgaleal drainage than the passive subdural drainage [10, 11, 16]. In addition, the recent systematic reviews and meta-analyses showed lower overall morbidity rate [9] and parenchymal injury rate [4, 17] following treatment with the active subgaleal drainage compared to the passive subdural drainage. One of the reasons behind the differences between studies could be the fact that complication rates following surgery for CSDH are generally low, and similarly, to our current study, many retrospective studies can be underpowered to show significant differences in complication rates between the postoperative drainage techniques. Moreover, complications might be underreported in retrospective studies especially in case of mild or asymptomatic complications such as small parenchymal injuries.

The main limitation of the present study was its retrospective design. Data was collected from the medical records, therefore some information could have been missing. The standard practice at our institution during both inclusion periods was drainage for 12–18 h postoperatively, while in the previous study [15], the reference center with the subgaleal cohort used the active subgaleal drainage for 24 h. Moreover, patients with passive subdural drainage were placed on bedrest for the duration of the drainage, while patients with the active subgaleal drainage could be mobilized freely. These differences could have perhaps contributed to the lack of difference in recurrence rates between the cohorts. In addition, only notes from the secondary and tertiary health care were available. As a result, the incidence of mild complications after surgery such as mild wound infections might be underreported in our study. Finally, unlike previous studies [1, 3] and a recent meta-analysis [13], we found no association between CSDH recurrence rate and haematoma diameter, density, or laterality. It was most likely due to the fact that the present study was underpowered to investigate multiple risk factors for haematoma recurrence.

## Conclusions

Conversion from the passive subdural to the active subgaleal drainage did not result in a clear reduction of reoperation rates for CSDH in our center.

## Data Availability

Data that support the findings of this study are available from the corresponding author, upon reasonable request and necessary ethical approval.
